# Effects of perinatal factors on sirtuin 3, 8-hydroxy-2′- deoxyguanosine, brain-derived neurotrophic factor and serotonin in cord blood and early breast milk: an observational study

**DOI:** 10.1186/s13006-020-00301-z

**Published:** 2020-06-17

**Authors:** Kata Nyárády, Réka Turai, Simone Funke, Erzsébet Györgyi, Alexandra Makai, Viktória Prémusz, József Bódis, Endre Sulyok

**Affiliations:** 1 Doctoral School of Health Sciences, Faculty of Health Sciences, University of Pécs, Pécs, Hungary; 2 Department of Obstetrics and Gynaecology, Medical School, University of Pécs, Pécs, Hungary; 3 Department of Laboratory Medicine, Medical School, University of Pécs, Ifjúság u. 13, Pécs, H-7624 Hungary; 4 MTA-PTE Human Reproduction Scientific Research Group, University of Pécs, Vörösmarty u. 4, Pécs, H-7621 Hungary

**Keywords:** Breast milk, Cord blood, Sirtuin, Serotonin, Brain-derived neurotrophic factor, 8-hydroxy-2′-deoxyguanosine

## Abstract

**Background:**

The profile of sirtuin 3 (SIRT3), 8-hydroxy-2′-deoxyguanosine (8-OHdG), brain-derived neurotrophic factor (BDNF) and serotonin (5-HT) in cord blood and in early breast milk was studied and it was related to perinatal factors. 5-HT and BDNF signalling systems have been claimed to play a critical role in intrauterine development, postnatal adaptation and lactation. Since prematurity and Caesarean birth are frequently associated with inflammation and related oxidative stress, an attempt was made to reveal the adaptive changes of the protective SIRT3 and the complex interplay among these bioactive components in cord blood and early breast milk.

**Methods:**

Three groups each consisting of 30 mothers were included in the study: mothers who underwent spontaneous vaginal birth at term (group I), Caesarean section at term (group II) and preterm birth (group III). Venous cord blood and early breast milk samples were collected for measuring the biomarkers. SIRT3, 8-OHdG, BDNF and 5-HT levels were determined by using commercially available ELISA kits.

**Results:**

It was demonstrated that cord blood levels of SIRT3, BDNF and 5-HT were markedly reduced whereas those of 8-OHdG were significantly elevated after preterm birth when compared with birth at term. The Caesarean section was associated with a moderate decrease in BDNF and 5-HT, however, both SIRT3 and 8-OHdG remained unaffected. Breast milk levels of all biomarkers studied proved to be independent of their corresponding cord blood concentrations. In response to preterm birth breast milk SIRT3, 8-OHdG and 5-HT increased significantly, while a drastic fall occurred in BDNF. A significant positive relationship was found of 5-HT with SIRT3 and 8-OHdG irrespective of the gestational age and the mode of delivery.

**Conclusions:**

It is suggested that the selected biomarkers in the breast milk mostly derive from local production by the mammary glands and 5-HT may have an essential role in the control of this process.

## Background

Breastfeeding is the optimal infants’ nutrition to achieve normal growth and development and to ensure short-term and long-term health outcomes [1–3]. It contains macro- and micronutrients [1, 3], factors deemed beneficial for immune protection [4], hormones [5, 6], growth factors [7] and an array of bioactive components [3]. Oligosaccharides unique for human milk have been proposed to play a key role in establishing the gut microbiota associated with lifelong consequences [8]. The composition of human milk, however, undergoes dynamic changes during lactation and the influences of several maternal, nutritional and environmental factors have been documented [1, 3].

Decades ago we carried out studies to reveal the involvement of prolactin and aldosterone in the control of sodium content of human milk with particular attention to the gestational and postnatal ages [9, 10]. Our interest in breast milk composition was renewed by current discoveries of the perinatal significance of some bioactive compounds including serotonin [5-HT] [11–13], brain-derived neurotrophic factor [BDNF] [14–16], the histone deacetylase sirtuins [SIRTs] [17, 18] and the oxidative stress marker, 8-hydroxy-2′-deoxyquanosine [8-OHdG] [19, 20].

To get some additional information on the adaptive changes in breast milk composition to perinatal events in the present study, we measured the levels of SIRT3, BDNF, 5-HT and 8-OHdG in cord blood and in early breast milk of mothers giving birth at term, vaginally or by Caesarean section, and of those delivering preterm neonates. 5-HT and BDNF signalling systems have been claimed to play a critical role in intrauterine development, postnatal adaptation and lactation. Since prematurity and Caesarean births are frequently associated with inflammation and related oxidative stress an attempt was made to reveal the adaptive changes of the protective SIRT3 and the complex interplay among these bioactive components in cord blood and early breast milk. Specifically, our study was undertaken a/ to define the impact of gestational age and the mode of delivery on cord blood serum and breast milk levels of these compounds, b/ to explore the relationship between their levels in the cord blood and breast milk and c/ to reveal the possible interactions of these bioactive components in the cord blood and also in the early milk separately.

It was expected that the results of this study may help to understand as to whether the bioactive components of breast milk investigated are exclusively the products of mammary glands or the maternal/placental circulation also contributes.

## Methods

Women were recruited during the third trimester of their pregnancy in their regular visit at the outpatient units of the Department of Obstetrics and Gynecology of the University of Pécs during the period of September 2016 and September 2017. Non-probability convenience sampling technique was used to enrol the participants irrespective of their primi- or multiparity.

Three groups each consisting of 30 mothers were included in the study. Mothers who underwent spontaneous vaginal delivery at term comprised Group I, those who had Caesarean section at term due to cephalo-pelvic disproportion, previous Caesarean section or imminent foetal asphyxia represented Group II, whereas Group III included mothers delivering preterm neonates at the gestational age of 30–36 weeks. Due to technical problems several samples [sample size and storage] 3, 1 and 2 patients were excluded from the groups of vaginal birth, Caesarean birth and preterm birth, respectively.

The main clinical characteristics of the patients are summarized in Table 1. In the case of term birth mothers had uneventful pregnancies without medication, none of them had a history of renal diseases, hypertension, diabetes mellitus, preeclampsia, infection, labour induction, premature rupture of foetal membranes, or mental health disorders. Epidural anaesthesia with Marcain [Actavis] or Bucain [Astra-Zeneca] was common; in the case of vaginal birth at term 12 patients, Caesarean section at term in 27 patients, and in the case of preterm birth 11 patients received this therapy. These drugs are local, non-opioid analgesics without apparent influence upon lactation. Uterine contractions were augmented with oxytocin at term birth either vaginally [*N* = 7] or Caesarean section [*N* = 6] and at preterm birth [*N* = 4]. Several mothers of the preterm infants received steroid prophylaxis [*N* = 17], beta-mimetic tocolysis [*N* = 3], antibiotic therapy [*N* = 7] and methyldopa for pregnancy-induced hypertension [*N* = 7]. Thirteen of the preterm infants were delivered by Caesarean section. Since the individual values of the biomarkers studied did not differ significantly from those born vaginally all preterm infants were considered as a single group irrespective of the mode of their birth. All preterm infants survived, however some of them needed respiratory support, antibiotics and intravenous fluid therapy. Breast milk feeding was implemented on the 1st day of life, and its volume was gradually increased as it was tolerated. Premature infants with gestational age of < 32–33 weeks in addition to their own mother’s milk received pooled human milk supplement. Blood was taken from the umbilical vein following the clamping of the cord. After the samples were drawn into syringes, all samples were immediately injected into native tubes in ice water. The serum was separated in a refrigerated centrifuge and stored at -20^o^ C degree until analysis.

**Table 1:** Basic characteristics of the patients (median with 25th and 75th centiles)

		Vaginal birth at term ***N*** = 27	Caesarean section at term ***N*** = 29	Preterm birth ***N*** = 28
**Gestational age**	(weeks)	39.0 (38.0;40.0)	39.0 (38.0;40.0)	34.0 (31.2;35.0)
**Birthweight**	(grams)	3410.0 (3110.0;3620.0)	3430.0 (3030.0;3880.0)	1940.0 (1525.0;2237.0)
**Apgar score**	(1 min)	9.0 (9.0;9.0)	9.0 (9.0;9.0)	9.0 (7.0;9.0)
**Apgar score**	(5 min)	10.0 (10.0;10.0)	10.0 (10.0;10.0)	10.0 (10.0;10.0)
**Medication**				
Epidural anaesthesia	*N* (%)	12 (44.4)	27 (87.1)	11 (39.3)
Oxytocin		7 (5.9)	6 (19.3)	4 (14.3)
Steroid prophylaxis				17 (60.7)
β-mimetic tocolysis				3 (10.7)
Antibiotics				7 (25.0)
Metyldopa				7 (25.0)
**Primiparae**		13 (48.1)	5 (17.2)	10 (35.7)

Milk samples were collected on the 2nd to 4th day after birth. The samples of about 1 ml were expressed by hand at the beginning and at the end of feedings. All specimens were pooled and stored at −20^o^ C until analysed. Milk samples were centrifuged at 4000×g for 10 min at 4 °C and the fat-poor infranatant fractions were collected from below the upper fat layer and used for ELISA. Our assays were not validated for breast milk, as we strictly followed the instructions prescribed by the manufacturers.

Laboratory measurements of sera and untreated, native milk samples were performed by using commercially available ELISA kits by IBL International GmbH Hamburg, Germany [5-HT, 8-OHdG], Cloud-Clone Corp. USA [SIRT3], and Ray Biotech. USA [BDNF]. The intra- and interassay coefficients of variation were < 10% and < 15%, respectively, for each kit.

Statistical analyses were performed using the 22.0 software of the SPSS [SPSS Inc. Chicago, IL, USA.]. Normality of data distribution was tested by using the Kolmogorov-Smirnov test. Based on the normality tests, we used non-parametric tests to compare the means of three groups [Kruskal-Wallis test] and to compare the means of two groups [Mann-Whitney U test and Wilcoxon test]. Spearman’s rank correlation was applied to examine the relationship between the different variables. Data were expressed as medians with 25th and 75th percentiles and a value of *p* < 0.05 was considered statistically significant.

## Results

Table 2 demonstrates SIRT3, 8-OHdG. BDNF and 5-HT levels in venous cord blood and breast milk after vaginal birth and Caesarean section at term as well as after preterm birth. It can be seen that serum SIRT3 levels were unaffected by the mode of term birth, although it was markedly reduced in preterm as compared to the full-term groups [vaginal birth and Caesarean section]. Interestingly, breast milk levels of SIRT3 much exceeded those measured in the cord blood, with significant difference in the case of Caesarean section and preterm birth. Furthermore, Caesarean section at term resulted in an elevation in breast milk SIRT3 levels without a significant increase after preterm birth.

**Table 2:** Cord blood and early breast milk levels of Sirtuin3 (Sirt3), 8-hydroxy-2-deoxyguanosine (8OHdG), brain-derived neurotrophic factor (BDNF) and serotonin (5HT) of mothers giving birth at term vaginally or by Caesarean section and those delivering preterm (median with 25th and 75th centiles)

	SIRT 3 (ng/ml)		8-OHdG (pg/ml)		BDNF (ng/ml)		5-HT (ng/ml)		Compared Groups
**I. Venous cord blood**		***p*** = 0.001** X^2^ = 13.61		***p*** < 0.001** X^2^ = 21.82		***p*** < 0.001** X^2^ = 38.55		*p* = 0.09 X^2^ = 4.91	1–2-3
1. Vaginal birth at term (*N* = 27)	0.27 (0.23;034)	*p* = 0.224 Z = -1.22	7.42 (0.05;14.41)	*p* = 0.54 Z = -0.61	77.94 (64.03;119.09)	***p*** = 0.007** Z = -2.72	90.96 (55.53;110.64)	*p* = 0.57 Z = -0.57	1–2
2. Caesarean section at term (*N* = 29)	0.24 (0.18;0.31)	***p*** = 0.02* Z = -2.41	8.41 (1.34,15.48)	***p*** < 0.001** Z = -3.68	57.22 (54.76;76.80)	***p*** < 0.001** Z = -4.68	73.55 (56.59;107.29)	*p* = 0.10 Z = -0.1.64	2–3
3. Preterm birth (*N* = 28)	0.20 (0.15;0.24)	***p*** < 0.001** Z = -3.62	21.10 (11.63;26.87)	***p*** < 0.001** Z = -4.28	36.22 (24.83;47.17)	***p*** < 0.001** Z = -5.40	58.39 (45.52;90.70)	***p*** = 0.04* Z = -2.07	1–3
**II. Breast milk**		*p* = 0.184 X^2^ = 3.39		***p*** = 0.01** X^2^ = 9.55		***p*** = 0.03* X^2^ = 7.23		***p*** = 0.02* X^2^ = 8.03	1–2-3
1. Vaginal birth at term (*N* = 27)	0.34 (0.17;1.27)	***p*** **= 0.06** Z = -1.94	13.29 (4.15;30.45)	*p* = 0.09 Z = -1.68	0.97 (0.76;1.11)	*p* = 0.68 Z = -0.42	14.69 (6.13;24.10)	*p* = 0.48 Z = -0.70	1–2
2. Caesarean section at term (*N* = 29)	1.72 (0.26;5.25)	*p* = 0.80 Z = -0.25	7.76 (3.20;17.6)	***p*** = 0.002** Z = -3.11	0.24 (0.20;0.94)	***p*** = 0.02* Z = -2.39	8.65 (3.43;23.35)	***p*** = 0.01* Z = -2.50	2–3
3. Preterm birth (*N* = 28)	2.19 (0.23;5.77)	*p* = 0.22 Z = -1.23	15.30 (10.85;35.97)	*p* = 0.24 Z = -1.17	0.03 (0.02;0.11)	***p*** = 0.008** Z = -2.65	25.82 (11.04;34.72)	***p*** = 0.02* Z = -2.25	1–3
**I-II. Cord blood and breast milk**									
1. Vaginal birth at term (*N* = 27)		*p* = 0.08 Z = -1.75		***p*** = 0.01* Z = -2.46		***p*** = 0.03* Z = -2.20		***p*** < 0.001** Z = -4.37	
2. Caesarean section at term (*N* = 29)		***p*** < 0.001** Z = -3.80		*p* = 0.61 Z = -0.51		***p*** = 0.02* Z = -2.37		***p*** < 0.001** Z = -4.76	
3. Preterm birth (*N* = 28)		***p*** < 0.001** Z = -4.00		*p* = 0.70 Z = -0.38		***p*** < 0.001** Z = -4.78		***p*** < 0.001** Z = -4.74	

Comparisons between three groups (1,2,3) by Kruskal-Wallis test, between two groups (1–2 or 2–3 or 1–3) by Mann-Whitney U test and between venous cord blood and breast milk (I-II.) were done by Wilcoxon test

**= *p* < 0.01, *= *p* < 0.05

No significant differences could be detected in 8-OHdG cord blood levels when infants were born at term either vaginally or by Caesarean section, however, its levels were doubled after preterm birth. Mothers giving birth at term vaginally had markedly elevated breast milk 8-OHdG levels but after Caesarean section or preterm birth breast milk 8-OHdG levels proved to comparable to their cord blood levels.

Following preterm birth, however, the breast milk 8-OHdG level significantly increased when compared with those obtained after Caesarean birth at term.

Cord blood BDNF was higher at term vaginal when compared to Caesarean birth, followed by even lower values in the preterm group. BDNF was present in the breast milk in minute amounts and its levels were significantly lower in the preterm than in the term groups irrespective of the mode of their birth. The pattern of cord blood 5-HT appeared to be similar to that of BDNF i.e. its serum levels in the Caesarean section group tended to be lower when compared to the vaginal group however, in the preterm birth group it reached a statistically significant further reduction compared to those giving birth vaginally at term. 5-HT in the breast milk could be measured in much lower concentrations, but contrary to BDNF its levels significantly increased in the group born prematurely. Characteristically, BDNF and 5-HT levels in breast milk were markedly depressed irrespective of gestation age or mode of birth.

When breast milk SIRT3, 8-OHdG, BDNF and 5-HT were analysed in relation to their cord blood levels no association could be established among the corresponding components. The potential interactions of these biologically active components of breast milk were also thoroughly examined. Distinctly, a significant positive association was demonstrated of 5-HT with SIRT3 and 8-OHdG in each group studied [vaginal birth at term *r* = 0.48, *p* = 0.014, and *r* = 0.78, *p* < 0.001, Caesarean section at term *r* = 0.40, *p* = 0.03 and *r* = 0.43, *p* = 0.02, preterm birth *r* = 0.54, *p* = 0.001 and *r* = 0.40, *p* = 0.02, respectively]. Moreover, SIRT3 was positively related to 8-OHdG; their respective values were for vaginal birth *r* = 0.41, *p* = 0.04, for Caesarean section *r* = 0.50, *p* = 0.006, and for preterm birth *r* = 0.40, *p* = 0.03. No interactions could be revealed for BDNF with any of the breast milk biomarkers investigated (Table 3).

**Table 3:** The interrelation between Sirtuin3, 8-hydroxy-2-deoxyguanosine, brain-derived neurotrophic factor and serotonin in early breast milk of mothers giving birth at term vaginally or by Caesarean section and those delivering preterm (Spearman’s rank correlation)

		Vaginal birth at term				Caesarean section at term				Preterm birth			
		SIRT3	8-OHdG	BDNF	5-HT	SIRT3	8-OHdG	BDNF	5-HT	SIRT3	8-OHdG	BDNF	5-HT
**Biomarkers in breast milk**													
SIRT3	R		0.41^*^	−0.20	0.48^*^		0.50^**^	0.05	0.40^*^		0.40^*^	−0.20	0.54^**^
	*p*		**0.04**	0.34	**0.01**		**0.006**	0.81	**0.03**		**0.03**	0.26	**0.001**
8-OHdG	R	0.41^*^		−0.29	0.78^**^	0.50^**^		−0.08	0.43^*^	0.40^*^		−0.26	0.40^*^
	*p*	**0.04**		0.16	**0.000**	**0.01**		0.67	**0.02**	**0.03**		0.15	**0.02**
BDNF	R	−0.20	−0.29		−0.34	0.05	−0.08		−0.17	− 0.20	− 0.26		− 0.26
	*p*	0.34	0.16		0.09	0.81	0.67		0.37	0.26	0.15		0.15
5HT	R	0.48^*^	0.78^**^	−0.34		0.40^*^	0.43^*^	−0.17		0.54^**^	0.40^*^	−0.26	
	*p*	**0.01**	**0.000**	0.09		**0.03**	**0.02**	0.37		**0.001**	**0.02**	0.15	

*SIRT3* Sirtuin 3, *8-OHdG* 8-hydroxy-2′-deoxyquanosine, *BDNF* Brain-derived neurotrophic factor, *5-HT* Serotonin

**= *p* < 0.01, *= *p* < 0.05

## Discussion

The present study demonstrated that cord blood levels of SIRT 3, BDNF and 5-HT were markedly reduced, whereas those of 8-OHdG were significantly elevated after preterm birth when compared with their levels following birth at term. Moreover, a Caesarean section at term was associated with a moderate decrease in cord blood BDNF and 5-HT yet cord blood levels of SIRT3 and 8-OHdG remained unaffected. Breast milk levels of BDNF and 5-HT proved to be much lower, 8-OHdG similar and SIRT3 increased when compared with the corresponding cord blood values. Mothers giving birth preterm experienced increased breast milk levels of SIRT 3, 8-OHdG and 5-HT, whereas a drastic fall occurred in their breast milk BDNF. Interestingly, the Caesarean section was associated with an increase of SIRT3, but it had no apparent influences on other compounds studied. A significant positive relationship was found of 5-HT with SIRT3 and 8-OHdG and also of SIRT3 with 8-OHdG, irrespective of the gestational age and mode of delivery.

As was expected, the profiling of 5-HT, SIRT3, BDNF and 8-OHdG in cord blood and breast milk allows us to assess their functional relevance during the perinatal period. 5-HT is a multifunctional intercellular signal molecule which has been claimed to play an important role in oocyte maturation, implantation, early embryonic/foetal development and postnatal adaptation [21, 22]. This notion is supported by the detection of major elements of 5-HT metabolism in gestational tissues [23, 24]. The source of 5-HT during development, however, remains a matter of scientific debate. Admittedly, there is evidence that it is supplied by the embryo/foetus, the placenta and by the maternal circulation. In a most recent study it has been proposed that 5-HT is released from maternal platelets, and then taken up by syncytiotrophoblasts and without being stored it is exported to the embryo/foetus [25, 26].

Postnatally, 5-HT is supplied in the breast milk as its levels were independent of those in the cord blood, we suggested that it mainly derives from the mammary glands. In fact, non-neuronal 5-HT emerged as a novel homeostatic regulator of the mammary gland and it has been shown to be involved in mammary gland involution, milk production, milk protein synthesis and cell apoptosis [26–28]. The production of 5-HT by the mammary gland has been substantiated in recent studies showing that peripartum exposure to fluoxetine [selective serotonin reuptake inhibitor; SSRI] selectively elevated the expression of tryptophan hydroxylase [Tph1] mRNA and the 5-HT content in the mammary gland [29]. Furthermore, the genetic disruption of Tph1 in the mammary gland during late gestation and lactation reduced its 5-HT content and also the serum levels of 5-HT [30]. Therefore, it appears therefore, that the mammary gland has an important role in 5-HT homeostasis during lactation and may coordinate the local secretion of several biologically active breast milk constituents including SIRT3. Our results revealed a close positive correlation of milk 5-HT with 8-OHdG and SIRT3, therefore, we suggest further functions for breast milk 5-HT.

8-OHdG has been established as a sensitive indicator of oxidative damage to DNA [31, 32] and in our study it was found to increase together with 5-HT and SIRT3 in preterm birth. SIRTs are a highly conserved protein family of NAD-dependent histone deacetylases which regulate cellular metabolism, redox state, stress signalling and confer protection for cell survival and genome stability [33, 34].

It is tempting to postulate that the similar response pattern of breast milk 8-OHdG, SIRT3 and 5-HT to the process of birth may be accounted for by the surge of pro-inflammatory cytokines and free oxygen radicals [35]. In this coordinated interplay 5-HT may have a prominent role in mediating the adaptive response of SIRT3 to potentially stressful events.

Unexpectedly, a clear dissociation was observed of BDNF from 5-HT in the breast milk which is inconsistent with the generally accepted concept of the positive feedback regulation of 5-HT and BDNF [36]. The reason for this finding is not evident, although one can assume that the stimulatory effects of 5-HT are overcome by the preterm birth and related pathologies including inflammatory reaction with or without apparent infection [37]. Consequently, BDNF concentration and supply is extremely reduced and may contribute to the developmental compromise of preterm neonates since BDNF is involved in the regulation of neuronal growth, survival and synaptic plasticity [38].

Our observations may have some clinical relevance. Pathophysiological events related to prematurity and birth by Caesarean section appear to depress BDNF in cord blood and early breast milk, may interfere with the feedforward regulation of BDNF-5-HT axis [36] and may result in neurodevelopmental delay. Furthermore, our results support the view that in mothers on SSRI therapy the low breast milk supply may be accounted for by the locally produced 5-HT [39, 40]. Consistent with this notion, in a most recent population-based cohort study Jordan et al. demonstrated that exposure to depression or to antidepressants particularly to high dose SSRIs during pregnancy increased the risks of exclusive formula feeding at 6–8 weeks [41].

Elevated 5-HT in early breast milk of mothers delivering preterm is assumed to activate the negative feedback loop of 5-HT and prolactin and to reduce the expression of prolactin-responsive genes in the mammary gland with the subsequent suppression of lactogenesis [40]. Mothers at risk of an elevated mammary gland 5-HT production, therefore, may benefit from the implementation of measures to enhance milk yields by restoring the 5-HT-prolactin balance.

The relatively small number of patients, the cross-sectional study design and convenience sampling are limitations. Furthermore, the assays we used were not validated for breast milk, thus analytical bias cannot be excluded. Prospective, longitudinal studies are currently underway to explore the impact of complex interactions of selected bioactive components on breast milk secretion once lactation is initiated and established.

## Conclusion

In conclusion, the present study provides suggestive evidences that some selected bioactive compounds [SIRT3, 5-HT, BDNF, 8-OHdG] in the breast milk mostly derive from local production by the mammary glands since their levels are independent of those in the cord blood. Furthermore, 5-HT appears to mediate the adaptive response of SIRT3 to preterm birth-related stressful events to attenuate their potentially adverse consequences.

## Data Availability

The dataset supporting the conclusions of this article is available from the corresponding author on reasonable request.

## Abbreviations

5-HT: Serotonin
BDNF: Brain-derived neurotrophic factor
SIRTs: Sirtuins
8-OHdG: 8-hydroxy-2′-deoxyquanosine
ELISA: Enzyme linked immunosorbent assays
NAD: Nicotinamide adenine dinucleotide
